# Geopolymer Foam with Low Thermal Conductivity Based on Industrial Waste

**DOI:** 10.3390/ma17246143

**Published:** 2024-12-16

**Authors:** Patrycja Bazan, Beata Figiela, Barbara Kozub, Michał Łach, Katarzyna Mróz, Mykola Melnychuk, Kinga Korniejenko

**Affiliations:** 1 Faculty of Materials Engineering and Physics, Cracow University of Technology, Jana Pawła II 37, 31-864 Cracow, Poland; patrycja.bazan@pk.edu.pl (P.B.); beata.figiela@pk.edu.pl (B.F.); barbara.kozub@pk.edu.pl (B.K.); michal.lach@pk.edu.pl (M.Ł.); 2 Faculty of Civil Engineering, Cracow University of Technology, Warszawska 24, 31-155 Cracow, Poland; katarzyna.mroz@pk.edu.pl; 3 Department of Materials Science, Lutsk National Technical University, Lvivska 75, 43000 Lutsk, Ukraine; m.melnychuk@lntu.edu.ua

**Keywords:** mine waste, geopolymer, foaming process, mechanical properties, thermal conductivity

## Abstract

Geopolymer materials are increasingly being considered as an alternative to environmentally damaging concrete based on Portland cement. The presented work analyzed waste from mines and waste incineration plants as potential precursors for producing geopolymer materials that could be used to make lightweight foamed geopolymers for insulation applications. The chemical and phase composition, radioactivity properties, and leachability of selected precursors were analyzed. Then, geopolymer materials were produced, and their strength properties were examined through compression and flexural tests. The results of the strength tests guided the material selection for foamed geopolymer materials. Next, geopolymer foams were foamed with hydrogen peroxide and aluminum powder. The produced foamed materials were subjected to strength and thermal conductivity tests. The results demonstrated the great potential of mine waste in the synthesis of geopolymers and the production of lightweight geopolymer foams with good insulating properties.

## 1. Introduction

Geopolymers are inorganic materials created by the alkaline activation of aluminosilicates [1,2]. The base materials to produce geopolymers are most often naturally occurring minerals from the group of silicates classified as clay minerals; namely, kaolin and metakaolin, rice husk ash, volcanic rock powders, fly ash, or slag are other precursor materials [3,4]. Nowadays, geopolymer research is gaining more and more interest, as it can be used as a cost-effective and economical alternative to organic polymers and inorganic cement in various applications, such as aviation, high-tech ceramics, thermal insulation foams, refractory building materials and adhesives, protective coatings, and hybrid organic-inorganic composites [5,6]. This budding interest is due to the physical, chemical, and mechanical properties of the geopolymers [7,8].

Research on geopolymer materials includes the analysis of the precursor selection, geopolymerization methods and modifications that tailor strength properties through additional components, time, annealing temperatures, and the type of activator [9,10]. The basic properties and advantages of geopolymers over conventional concrete are their high compressive strength, thermal resistance, non-flammability, reduced energy demand, low carbon dioxide emission, increased durability, better workability, and reduced shrinkage [11,12]. Moreover, some geopolymers, especially those based on metakaolin or fly ash class F, have limited predisposition to cracking. Because of that, these materials are used in civil engineering for soil stabilization, slabs, insulation, cubes, etc. [13,14]. In addition, these materials are characterized by low permeability and chemical stability. Therefore, geopolymers are also considered advantageous materials for use in different civil engineering applications, including in marine infrastructure [15,16].

Thermal resistance is a critical property of geopolymers. A comparison of the fire resistance of conventional concrete and a fly ash-based geopolymer indicated that the concrete was limited to a temperature of 800 °C, beyond which the material undergoes intensive cracking. In contrast, at the same temperature, the geopolymer was characterized by small and few cracks [17,18,19].

The current literature on geopolymer materials focuses on solid materials, while scientific reports on geopolymer materials in the foamed form are receiving increased attention [20,21]. Foamed geopolymer materials are characterized by relatively low thermal conductivities. Similarly, the low weight of foamed geopolymers is considered regarding insulation materials, partitions, and void fillings [20,22,23]. Foamed geopolymer is obtained by chemical or mechanical foaming [24,25]. Research on foamed geopolymers has shown that these materials achieve thermal conductivities ~50% lower than concrete, ranging from 0.072–0.48 W/(m·K) [25,26,27]. The thermal properties of geopolymers are influenced by parameters such as size, distribution, pore shape, type and content of the foaming agent, type and content of the binder, activator content, temperature, and method of foaming [28,29,30].

The presented work demonstrates the enormous potential of geopolymer materials due to their advantageous properties. The indispensable advantages of geopolymer materials related to environmental protection, reducing the negative environmental impact of concrete based on Portland cement, and meeting commitments associated with the circular economy were highlighted. The work aims to synthesize a geopolymer foam with low thermal conductivity based on industrial waste as an innovative material for the circular economy. The work was divided into two stages. The first stage concerns the selection and preparation of waste materials (coal shale, fly ash, post-processing waste) for use as raw materials in the synthesis of inorganic polymers. In the second stage, the synthesis of waste-based geopolymers and the development of the foaming process were studied. Various foaming methods, in particular aluminum powder, hydrogen peroxide, and mixed methods, were utilized to prepare the foamed geopolymers. The presented research will also contribute to the knowledge of the risks associated with waste materials disposal. According to the circular economy, the methods of immobilization and the use of by-products stored in landfills to develop building materials are critical.

## 2. Materials and Methods

### 2.1. Materials

The first stage of research was the preparation of materials based on waste sources (fly ash, mining coal waste, and post-processing waste), and the following list presents applied materials:

- Ash from the municipal waste treatment plant in Białystok—a waste incineration plant that processes ~15 tons of municipal waste per hour [31].
- Fly ash from the combined heat and power plant in Skawina—this plant is a producer of electricity produced in combination with heat and is also a supplier of process steam, drinking water, and industrial water [31].
- Mining waste from the Tauron Wydobycie S.A. mine Zakład Górniczy Sobieski—a hard coal mine located in Jaworzno. The type of material used is flame coal.
- Mining waste from the Murcki-Staszic hard coal mine in Katowice—the exploited coal is described as low quality due to a large amount of sulfur and a small amount of chlorine.
- Mining waste from the Wieczorek II and Wujek hard coal mines—a mine located in Katowice.

The selected ashes showed sufficient reactivity in the synthesis process and did not require an additional calcination process. This was confirmed by the previous research [31]. Therefore, the activation process using mechanical methods was carried out using mine waste. Due to their large dimensions, the first stage of work with coal shale was fragmentation into smaller particles by crushing. Then, each of the crushed materials was ground in a wheel mill to obtain particles smaller than 200 μm. After each grinding process, the obtained type of fraction was examined. First, the particles larger than 200 μm were crushed in an automatic grinder on a sieve with a mesh size of 0.7 mm and then a 200 μm mesh sieve. In total, 4 kg of ground powders for each raw material were obtained. Then, the powders were heated (calcined) in a laboratory furnace at 700 °C for 24 h to facilitate alkaline activation, allowing for the appropriate microstructure.

The thermal treatment of aluminosilicate materials causes changes in their structure, resulting from the growth of the amorphous phase. Therefore, the mechanical strength of alkali-activated binders depends on the structural conditions of the aluminosilicate materials. Scientific research has shown that higher mechanical strength results from using calcined materials, such as metakaolin, or materials produced as by-products of high-temperature fly ash processes or blast furnace slag [32,33]. As with the pozzolanic reactivity, the alkali activation reactivity depends on the amorphous content of silica and aluminum. The reactivity is related to the structure of the material and increases with greater amorphous content [34].

### 2.2. Sample Preparation

Geopolymers were prepared using the sodium promoter and six waste materials acting as precursors. The activation process was performed using a 10 M solution of sodium hydroxide (NaOH) in combination with a solution of sodium silicate (and an aqueous solution of sodium silicate R-145 with a module of 2.5 molar and a density of approximately 1.45 g/cm^3^) in the proportion of 1:2, which is the most used hydroxide activator in the synthesis of geopolymers. Furthermore, this solution is also the cheapest and most widely available of alkali hydroxides. The solution was mixed and allowed to stabilize to temperature and equilibrium concentrations, which took about 2 h, and then the solution was mixed with the precursors (in six compositions obtained from waste materials that are presented in Table 1). After obtaining a homogeneous mass with a thick, plastic consistency, the mixtures were transferred to molds and then vibrated using a vibrating table. The formed geopolymers were heated in a laboratory dryer (SLW 750 STD, POL-EKO-APARATURA, Wodzisław Śląski, Poland) for 24 h at 75 °C under atmospheric pressure. After 24 h, the samples were removed from the molds. The samples were designated according to the places and origin of the raw materials—Table 1.

A foaming process was carried out on selected precursors to obtain lightweight geopolymer materials for insulation purposes. The foaming process was carried out by adding hydrogen peroxide 36% and aluminum powder to the geopolymer paste (Table 2).

The detailed amounts are presented in Section 3.6.

### 2.3. Methods

The mineral composition of the geopolymer precursors was analyzed with X-ray diffraction (XRD) (PANalytical, Almelo, The Netherlands) using the powder X-ray method (Debye-Scherrer). Phase analysis for all materials was performed on the PANalytical AERIS X-ray diffractometer using Cu-Kα radiation. The obtained distance between the planes was used to identify the phases. X-ray analysis was performed using the HighScore Plus software (v5.1) and the PDF4 + crystallographic database. Particle size analysis was made using the laser diffraction method on the Anton Paar PSA 1190 LD Graz particle analyzer (Anton Paar GmbH, Graz, Austria). Leachability and radioactivity tests were also commissioned for all analyzed materials. A leachability test was performed by GIG (Katowice, Poland). It was made according to PN EN 12457-2:2006 standard. Radioactivity tests were performed at the accredited AP Geotechnika Laboratory (Siemianowice Śląskie, Poland) following the PN EN 12457-2:2006 standard. Fourier-transform infrared spectroscopy (FT-IR) analysis of bonding in the material was carried out with the use Spektromer FT-IR Nicolet 6700 (Nicolet, Klapálkova, Czech Republic). It was performed by the Department of Medical Physics of the Jagiellonian University. Microscopic observations and analysis of the chemical composition in the micro-regions were performed using scanning electron microscopy (SEM, JEOL JSN5510LV) with an energy dispersive spectrometer (EDS), (JEOL, Tokyo, Japan). The density was determined using a geometric method. Density was determined as a ratio of mass to volume. Volume was calculated based on the arithmetic means of the measurements for five cuboidal samples for compressive strength tests for each of the analyzed compositions. Strength tests of the geopolymer materials were performed on cubic samples (50 mm × 50 mm × 50 mm) to determine compressive strength (PN-EN 12390-3:2019 Testing of concrete—Part 3: Compressive strength of test specimens). Flexural strength tests (PN-EN 12390-5:2019 Testing hardened concrete—Part 5: Flexural strength of test specimens) were carried out on prismatic samples (50 mm × 50 mm × 200 mm) with a 150 mm distance between the support points. Strength tests were carried out on a Matest 3000 kN testing machine (Matest, Treviolo, Italy); the speed of the tests was 0.05 MPa/s. The thermal conductivity was tested on the HFM 446 plate apparatus (Wittelsbacherstrasse, Germany). The average temperature of testing was 10 °C. The temperature of the upper plate was 20 °C and the lower plate was 0 °C. The distance between the plates was equal to the plate thickness. All tests were performed after 28 days of conditioning the geopolymeric samples at room temperature.

## 3. Results and Discussion

### 3.1. Summary XRD Analyses Performed and Results

**Figure 1:**
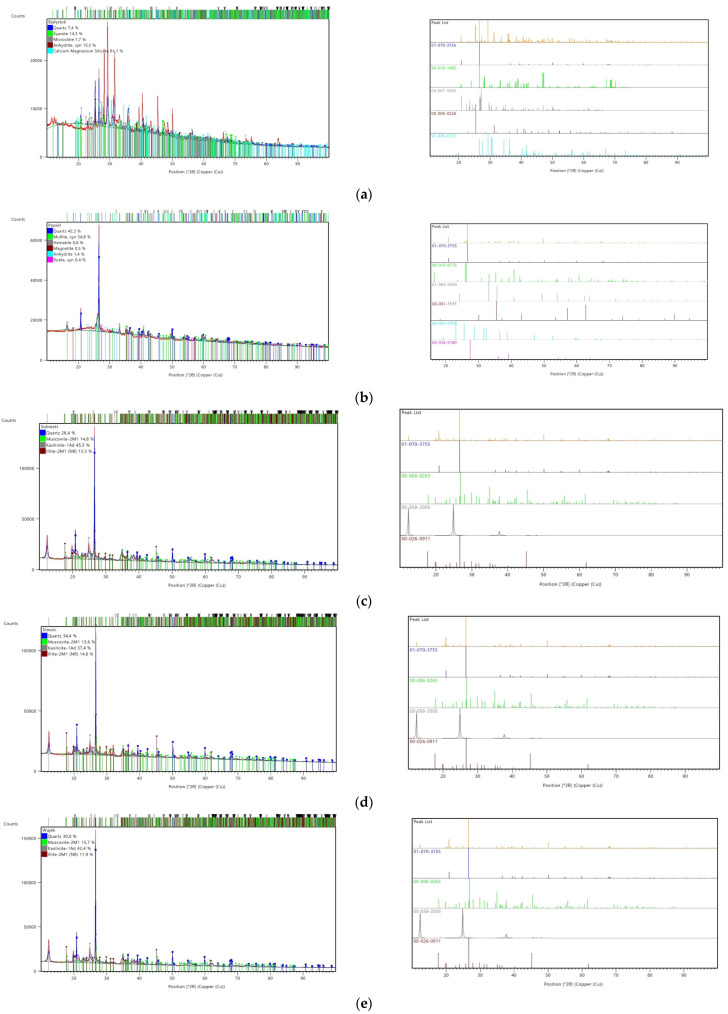

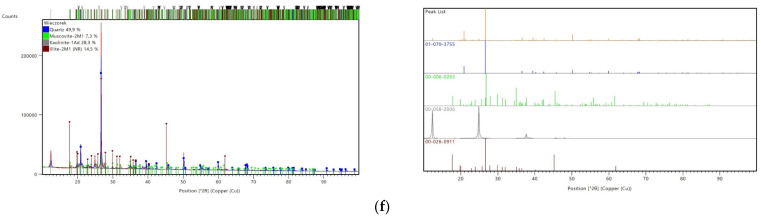
XRD patterns for raw materials (**a**) Białystok ash, (**b**) Skawina fly ash, (**c**) Sobieski, (**d**) Staszic, (**e**) Wujek, (**f**) Wieczorek.

Although geopolymers have an amorphous phase, the XRD technique helps analyze their chain formation. Figure 1 summarizes the obtained patterns from XRD analysis.

Table 3 summarizes the percentages of individually identified phases for all tested materials to enable comparative analysis.

The analysis of the XRD techniques demonstrated that the tested carbon shales contain layered silicates such as quartz, kaolinite, muscovite, and illite. Waste material obtained from Katowice’s Wieczorek II hard coal mine exhibited the highest quartz content (49.9%) followed by the Skawina mine (42.3%). However, material from the Staszic coal mine and Wujek contained almost equal silicon content. The greatest concentration of kaolinite, with the lowest range of illite, was exhibited in material from the Sobieski mine.

### 3.2. Summary of the Microscopic Examinations and EDS Analysis

The shapes and sizes of the raw materials’ particles depend on many factors. The particle size of the tested materials ranged from 5–200 µm [31]. The smallest, regular-shaped particles were found in fly ash from the Skawina CHP Plant, which contained few impurities (Table 4). The remaining materials were characterized by a large particle size distribution. The chemical composition of the materials is characterized by the content of silicon, aluminum, potassium, titanium, sulfur, chlorine, sodium, calcium, and magnesium compounds. Minerals rich in iron, titanium, sulfur, phosphorus, boron, alkali metal, and alkaline earth metal cations are common, and their distribution in the material is very variable. Each coal particle may contain different amounts of inorganic substances, and thus the resulting ash may be highly heterogeneous.

**Figure 2:**
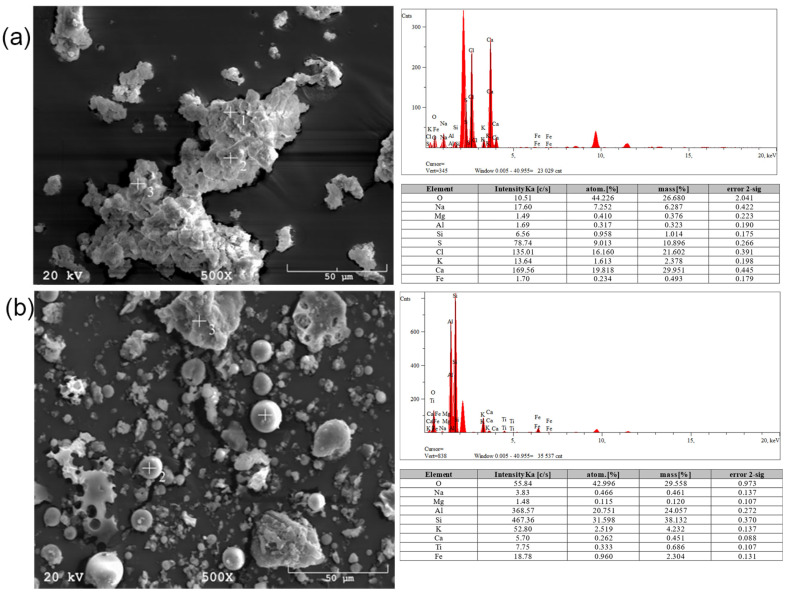
SEM micrographs and EDS analyses for (**a**) Białystok ash and (**b**) Skawina fly ash.

**Figure 3:**
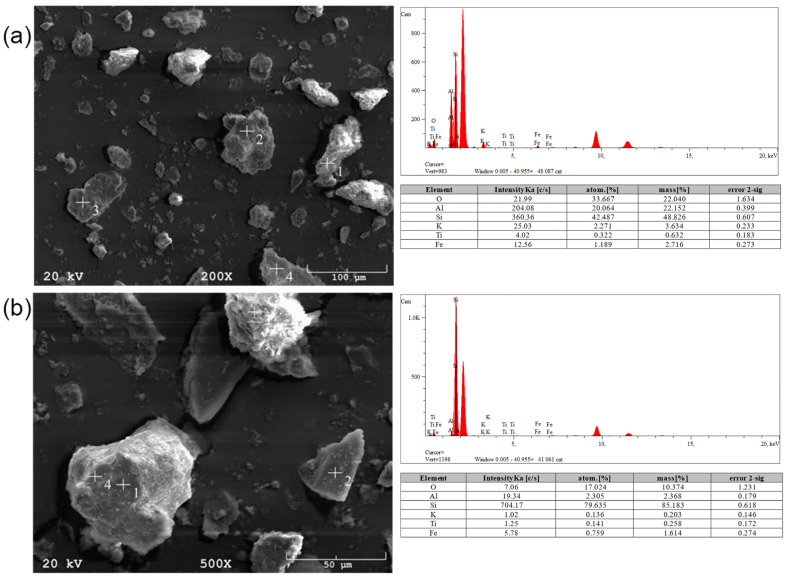
SEM micrographs and EDS analyses for (**a**) Sobieski and (**b**) Staszic coal shell.

**Figure 4:**
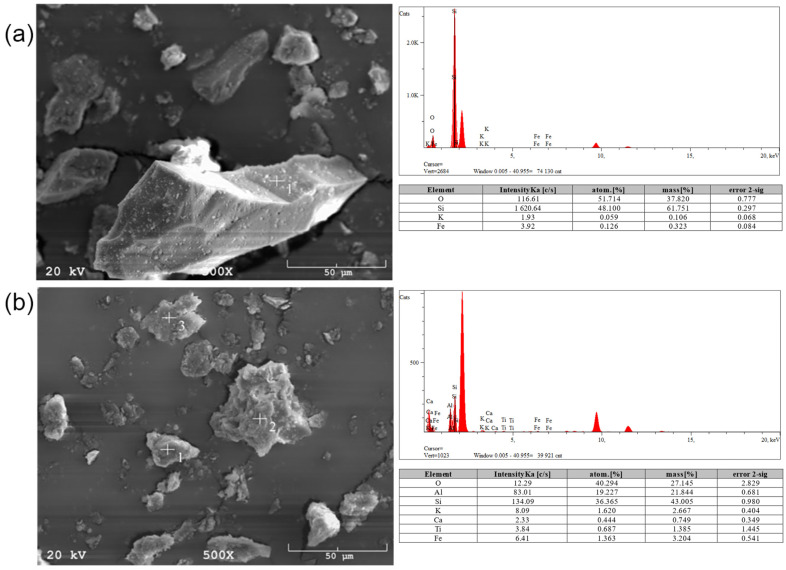
SEM micrographs and EDS analyses for (**a**) Wieczorek and (**b**) Wujek coal shell.

The titanium present in the ashes can affect the formability of the material. Titanium is present as an impurity in kaolin and other clays. Moreover, titanium is an excellent nucleating agent for crystallization. Other particles may also be present in the coal fly ash, including reactive calcium silicate phases similar to those in Portland cement. These particles are the result of cement that fills the pores in the carbon. The presence of calcium in large amounts can interfere with the polymerization process and change the microstructure [34]. However, there are reports of a positive effect of calcium on the geopolymerization process. Previous research has shown that fly ash with a high concentration of calcium is suitable as a source material for producing good-quality geopolymer materials [35,36]. The calcium in the fly ash creates the hydrated phase of calcium silicate (C-S-H). The coexistence of this phase with geopolymer gel positively affects the strength properties of the obtained geopolymer materials. In addition, the high calcium content causes calcium atoms to bind in the geopolymer lattice and act as a charge-balancing cation [37,38]. The microscopic images and EDS analyses are shown in Figure 2, Figure 3 and Figure 4.

### 3.3. Analysis of Radioactivity and Leachability

Leachability and radioactivity tests were carried out for all analyzed materials (Table 5 and Table 6).

Regarding the interpretation of the radioactivity results, the important values are f_1_ and f_2_. The most restrictive requirements are for the buildings to permanently house people and animals—for example, residential buildings. In this case, the values of f_1_ and f_2_ have to fulfill the following requirements: f_1_ < 1.2. and f_2_ < 240 Bq/kg. All materials meet these requirements.

The leachability and radioactivity tests for all studied materials showed compliance with the applicable standards and regulations [39,40]. Therefore, the materials can be safely used for construction products.

### 3.4. Summary FTIR Analyses Performed and Results

**Figure 5:**
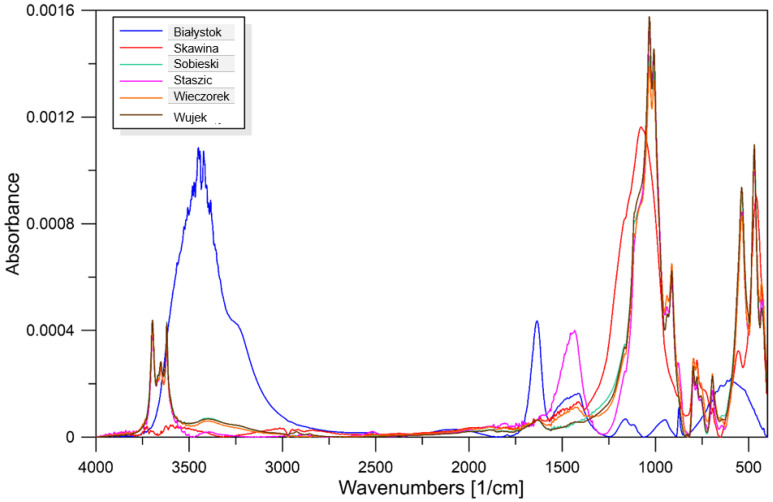
FTIR micrographs of the geopolymers made from the obtained waste materials.

Infrared spectroscopy using attenuated total reflectance is a technique widely used to detect the presence of water and the soluble silicate ratio in geopolymers. Some of the infrared bands found in materials and geopolymers include an intense band located in the range of 1250–950 cm^−1^, which corresponds to the asymmetric stretching of Si-O-R * (where R * may represent Si o Al) [41,42,43]. However, these vibrations could be found in the infrared spectra of aluminosilicates and aluminum. Some authors [44,45] suggest that the shift of this band towards lower or higher frequencies indicates the successful completion of the polycondensation reaction. The band located in the range of 3600–2700 cm^−1^ corresponds to the vibrations of -OH groups due to the presence of hydrogen bonds. Some authors reported weak Si-O-Al vibrations in the range of 460–470 cm^−1^ or 610–620 cm^−1^ [41,44]. The band located in the range of 1425–1450 cm^−1^ is associated with -CO_3_ and appears as a result of atmospheric carbonation occurring during sample synthesis [46]. The detailed results are presented in Figure 5.

### 3.5. Strength Tests of Manufactured Geopolymers

**Figure 6:**
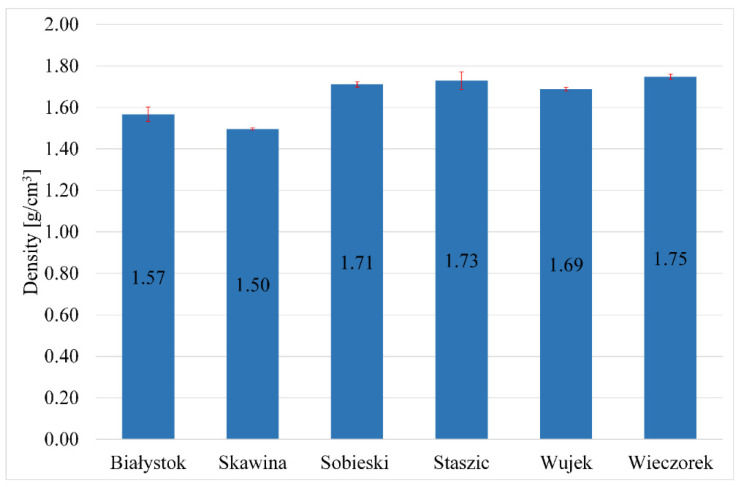
Densities of the geopolymers made from the obtained waste materials.

Before carrying out the strength tests, the density of the samples was measured using the geometric method. Figure 6 shows the average density values.

**Figure 7:**
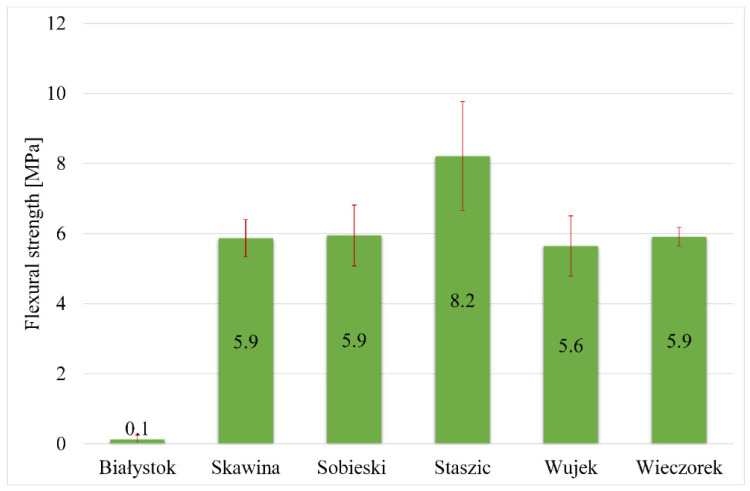
Flexural strength of the geopolymers made from the obtained waste materials.

**Figure 8:**
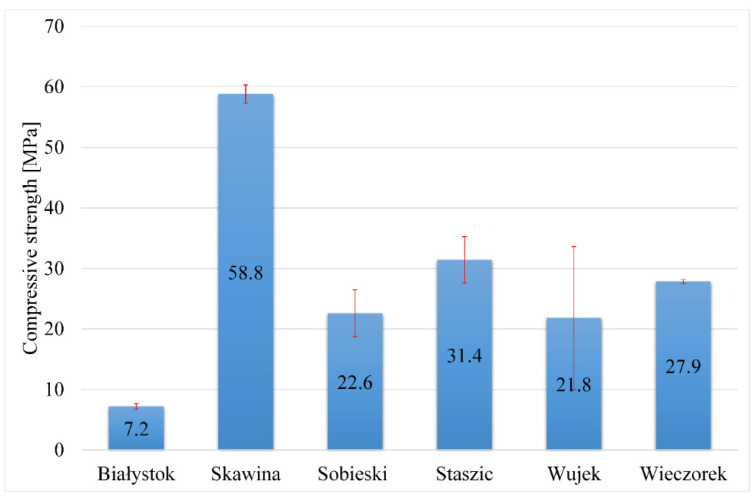
Compressive strength of the geopolymers made from the obtained waste materials.

The density should correlate with the compressive and flexural strength of geopolymers. In the case of the tested samples, this tendency is slight. This tendency is more visible in the case of foamed materials, where more voids appear. Samples prepared using materials obtained from the mine have a density range of 1.69–1.75 g/cm^3^. The obtained flexural and compressive strength results were presented graphically (Figure 7 and Figure 8).

The Skawina sample with Skawina fly ash content exhibited the greatest compressive strength (58.8 MPa). The Staszic sample had a compressive strength nearly two times lower (31.4 MPa) than the Skawina sample. The Wieczorek sample had a slightly weaker compressive strength (27.9 MPa) than the Staszic sample. The most insufficient compressive strength was for the sample prepared using waste material from Białystok (7.2 MPa).

In the case of flexural tests, the highest flexural strength was obtained for the Staszic sample (8.2 MPa). The remaining samples, comprised of coal shale and Skawina fly ash, exhibited similar flexural strength values of ~5.9 MPa. Samples from the waste incineration plant in Białystok had the lowest flexural strength (similar to the compression test results).

Summarizing the obtained compressive and flexural strength results, the highest values were obtained for geopolymers based on waste material from the Staszic and Skawina mine, which had a high content of quartz and kaolinite. On the other hand, samples obtained using the material from the waste incineration plant in Białystok demonstrated significantly worse results. It is worth noting that some studies of geopolymer materials indicate that even a small amount of quartz may positively affect mechanical properties. In contrast, the presence of other minerals may have opposite effects. For example, illite causes changes in geopolymer morphology and leaching behavior [47].

The results of the remaining samples, which are not significantly different, are the starting point for future studies regarding these materials, e.g., in variable proportions. The main difference between geopolymer concrete and Portland concrete is the binder. The silicon and aluminum oxides in the low calcium fly ash react with the alkaline liquid to form a geopolymer paste that binds loose, coarse aggregates, fine aggregates, and other unreacted materials to form geopolymer concrete. As with Portland concrete, coarse and fine aggregates account for approximately 75–80% of the weight of geopolymer concrete. This component of geopolymer concrete mixes can be designed using the tools currently available for Portland concrete. The compressive strength and workability of geopolymer concrete are influenced by the proportions and properties of the constituent materials composed by the geopolymer paste [34].

### 3.6. Microstructure Analysis of Manufactured Geopolymers

**Figure 9:**
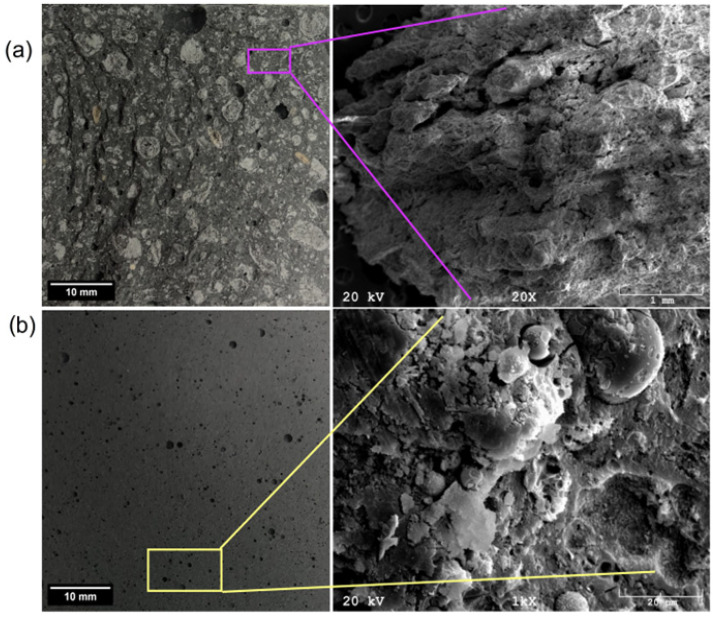
Optical and SEM images of (**a**) Białystok and (**b**) Skawina geopolymer samples.

Figure 9a presents a geopolymer based on Białystok ash. In the case of this material, at higher magnifications, polygonal pores were observed. The material’s structure was brittle and segmented, resulting in deficient strength properties. EDS analysis showed high calcium, sodium, and potassium content, and low levels of sulfur, silicon, aluminum, chromium, iron, nickel, and copper. In the micrographs of geopolymers based on Skawina fly ash (Figure 9b), a compact structure and visible pores with a maximum size of about 0.3 mm can be observed. However, most of the pores ranged from 10–30 µm. The structure is amorphous. The characteristic fine-grained structure of zeolites and undiluted ash particles were visible in the cavities. EDS analysis revealed the presence of elements such as silicon, aluminum, calcium, sodium, potassium, iron, and carbon.

**Figure 10:**
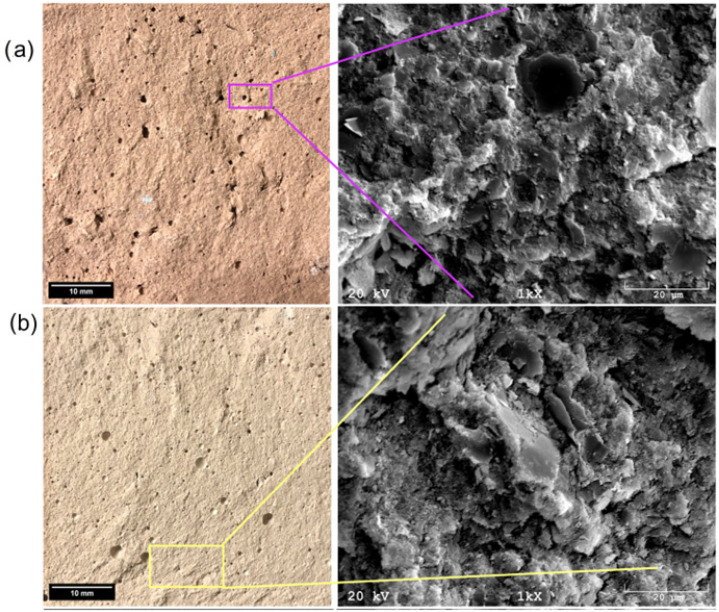
Optical and SEM images of (**a**) Sobieski and (**b**) Staszic geopolymer samples.

Another tested material was a geopolymer based on coal shale from the Sobieski mine (Figure 10a). The pore size was ~25 µm. The structure is brittle but relatively contained, with no visible cracks. Undiluted particles 10–20 µm in size were also observed. EDS analysis showed the presence of silicon, aluminum, calcium, magnesium, and iron compounds. There is a lack of significant changes compared to raw materials because light elements such as sodium are not properly detected in this method. It is also worth mentioning that EDS analysis is a qualitative method, so it does not give representative results according to the amount of element in the whole material volume. The microstructure of the geopolymer made from the Staszic mine (Figure 10b) is significantly different compared to previous materials. A significant variation in the size of the structure can be noticed, ranging from a few micrometers to several dozen. Large amorphous areas and very fine crystalline areas are presented. The pore size is ~50 µm. EDS analysis showed the presence of silicon, aluminum, calcium, chlorine, iron, and potassium compounds.

**Figure 11:**
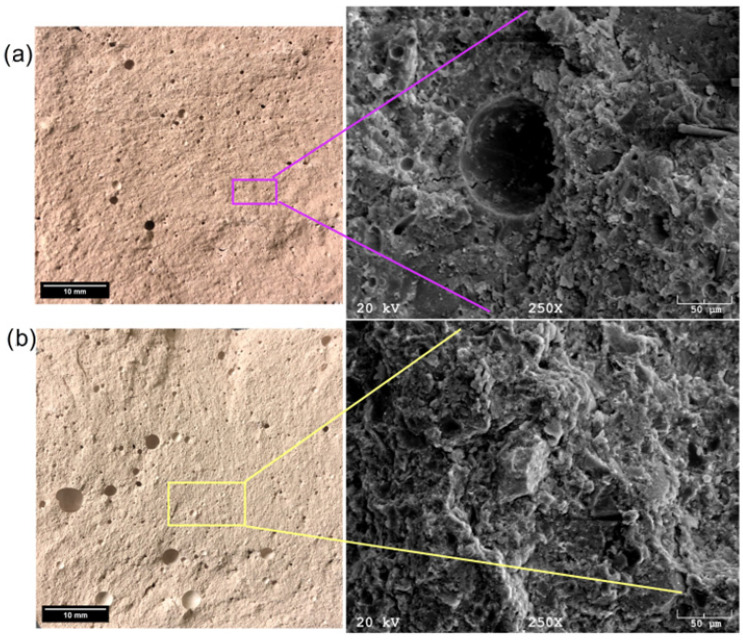
Optical and SEM images of (**a**) Wieczorek and (**b**) Wujek geopolymer samples.

Geopolymers based on slate obtained from the Wieczorek mine (Figure 11a) are characterized by significant porosity, ranging from tiny pores in the range of 2–3 µm to pores as large as 0.5 mm. The EDS analysis identified silicon, aluminum, sodium, calcium, potassium, chlorine, and iron compounds, as well as trace amounts of carbon. The last tested material was a geopolymer based on ash from the Wujek mine (Figure 11b). The microstructure analysis showed the presence of pores in the 50 µm range. Again, this material consists of silicon, aluminum, calcium, chlorine, iron, and potassium compounds.

The present research facilitated material selection for future works connected with the forming process.

### 3.7. Development of the Foaming Process of Waste-Based Geopolymers

The final stage of the presented results was the preparation of foamed materials using two foaming agents: aluminum powder (powder, max. particle size 60 µm, purity 99.9%, Sigma-Aldrich, St. Louis, MO, USA) and hydrogen peroxide (perhydrol 36%, Biomus, Lublin, Poland). Polyglycolic acid (Sigma-Aldrich) and cellulose (Sigma-Aldrich) were added as stabilizers. To prepare the samples, the precursors were mixed with the alkaline activator (NaOH) for 5 min. Next, foaming agents and the stabilizer were added, mixed for 30 s, and left for foaming. The list of produced materials is shown in Table 2. The properties of the foams are related to the introduced foaming agent, but this is not the only factor affecting the quality of the materials. In the production of samples, the process of mixing the materials, the concentration of additives used, and the temperature of the geopolymerization process are also important factors. Blowing agents such as aluminum oxide or hydrogen peroxide, in combination with calcium oxides present in the matrix and binder, emit hydrogen, which gives porosity to the geopolymer structure. During this reaction, hydrated calcium silicate is released, while hydrogen, being lighter than air, escapes from the geopolymer mass, increasing the mold volume. After some time, the hydrogen completely escapes from the mass and is replaced with air, filling the spaces (pores) of the foamed geopolymer. Aluminum powder reacts with calcium oxides and calcium hydroxides. In turn, hydrogen peroxide (perhydrol) also reacts with calcium chlorides. Table 2 lists the composition of the manufactured materials.

In this part, two materials based on mine tailings have been selected. We rejected the material from ashes because the Bialystok has very poor results, and data about foaming fly ash class F (such as Skawina) are relatively common. There are not many publications that investigate the foaming process for mine tailing-based geopolymers. These particular mine tailings have been selected because of their availability and reasonable properties gained on the previous steps presented in this article.

Strength parameters were assessed for the obtained samples, which are presented in Table 7. The lowest densities were observed in samples foamed with hydrogen peroxide. Conversely, the optimal properties for compressive strength were observed in samples foamed with aluminum powder.

There is a correlation between the obtained strength properties and the sample density. The compressive strength is increasing together with material density. Additionally, thermal conductivity tests were carried out for selected samples. The results for geopolymer foams based on mine wastes are presented in Table 8. The lowest thermal conductivity was found in the material based on mine wastes foamed with hydrogen peroxide, while materials foamed with aluminum powder were characterized by a relatively high thermal conductivity. Upon visual inspection of the obtained samples, the materials foamed with aluminum powder were characterized by lower porosity and lower volumetric compactness of porosity. The results of thermal conductivity confirm the conclusions put forward by other researchers that the greater the number of pores containing air, the lower the density of the material, and thus the lower the value of thermal conductivity [48].

## 4. Conclusions

The work aimed to select and prepare waste materials (mine waste, coal shale, fly ash, post-process waste) to be used as raw materials in the synthesis of inorganic polymers and subsequently the synthesis of waste-based geopolymers and the development of the foaming process. Hydrogen peroxide and aluminum powder were selected for the foaming process of geopolymer materials. The results indicated that geopolymer foams may successfully act as an insulation material. Research on geopolymer materials is a rapidly developing field of material science. Geopolymer materials are characterized by low thermal conductivities, good strength properties, and low densities.

- Mineralogical composition showed a slightly different composition of ashes and main tailings. In the case of main tailings, their composition was similar with different proportions of layered silicates such as quartz, kaolinite, muscovite, and illite.
- The chemical composition demonstrated that selected waste and ashes could be used as the base material to produce geopolymers, especially because of a sufficient amount of silica and alumina.
- The leachability and radioactivity tests for all analyzed materials were compliant with the applicable standards.
- Samples prepared using materials obtained from the mine have a density range of 1.69–1.75 g/cm^3^. The density of samples made from industrial-waste ashes was slightly lower.
- The highest flexural strength was obtained for the Staszic sample (8.2 MPa). The remaining samples, comprised of coal shale and Skawina fly ash, exhibited similar flexural strength values of ~5.9 MPa. Samples from the waste incineration plant in Białystok had the lowest flexural strength.
- The Skawina sample with Skawina fly ash content exhibited the greatest compressive strength (58.8 MPa). The Staszic sample had a compressive strength nearly two times lower (31.4 MPa) than the Skawina sample. The Wieczorek sample had a slightly weaker compressive strength (27.9 MPa) than the Staszic sample. The most insufficient compressive strength was for the sample prepared using waste material from Białystok (7.2 MPa). The waste materials from Białystok do not have enough mechanical strength for building applications.
- Thermal conductivity tests demonstrated that the optimal foaming agent was hydrogen peroxide, which ensured thermal conductivity (0.11609 W/(m∙K)). However, the foamed materials were characterized by a relatively low compressive strength (0.69 MPa). Therefore, future research will emphasize the selection of the most promising fillers, e.g., fibers and particles, and the manufacturing of a foamed composite material.

## Figures and Tables

**Table 1:** The designations of the tested samples—description of the selected sample index regarding the origin of the materials used in the production of geopolymers.

No	Designation	Origin of the Materials
1	Białystok	Municipal Waste Disposal Plant, Białystok
2	Skawina	Skawina Heat and Power Plant, Skawina
3	Sobieski	Tauron Wydobycie S.A. Z G. Sobieski, Jaworzno
4	Staszic	Polska Grupa Górnicza S.A. Branch of KWK Murcki-Staszic, Katowice
5	Wujek	Polska Grupa Górnicza S.A. KWK Wujek branch, Katowice
6	Wieczorek	SRK S.A. in Bytom, Branch of KWK “Wieczorek II”, Katowice

**Table 2:** List of components investigated in the manufactured panels for future research.

Index	Solution	Foaming Agent	Stabilizer
Wieczorek	NaOH 10M	4.5 g Al	-
		1.5 g Al	
		30 mL Perhydrol 36%	20 mL polyglycol
		30 mL Perhydrol 36%	5 g cellulose
Staszic		4.5 g Al	
		30 mL Perhydrol 36%	20 mL polyglycol
		30 mL Perhydrol 36%	5 g cellulose

**Table 3:** Summary of the XRD phase analysis for all tested materials.

Index	Percentage [%]								
	Quartz SiO_2_	Muscovite-2M1 KAl_2_(Si_3_Al)O_10_(OH,F)_2_			Kaolinite-1Ad Al_2_Si_2_O_5_(OH)_4_		Illite-2M1 (K,H_3_O)Al_2_Si_3_AlO_10_(OH)_2_		
Sobieski	26.4	14.8			45.5		13.3		
Staszic	34.4	13.6			37.4		14.6		
Wieczorek	49.9	7.3			28.3		14.5		
Wujek	30.0	15.7			42.4		11.9		
Index	Percentage [%]								
	Quartz SiO_2_		Cyanite Al_2_SiO_5_	Microcline KAlSi_3_O_8_		Anhydrite CaSO_4_		Diopside MgCaSi_2_O_6_	
Białystok	7.4		14.3	1.7		15.5		61.1	
Skawina	Percentage [%]								
	Quartz SiO_2_		Mullite Al_6_Si_2_O_13_	Hematite Fe_2_O_3_		Magnetite Fe_3_O_4_		Anhydrite CaSO_4_	Rutile TiO_2_
	42.3		54.8	0.6		0.5		1.4	0.4

**Table 4:** Summary of the particle size and chemical composition of the tested materials.

Index	Particle Size, [μm]	Chemical Composition
Białystok	5–100	Compounds of calcium, chlorine, sulfur, sodium, potassium, and silicon
Skawina	<20	Compounds of silicon, aluminum, calcium, and iron
Sobieski	20–100	Compounds of silicon, aluminum, potassium, iron, and titanium
Staszic	2–100	Compounds of silicon, aluminum, potassium, and iron
Wujek	10–70	Compounds of silicon, aluminum, potassium, and iron
Wieczorek	30–200	Compounds of silicon, aluminum, potassium, iron, chlorine, calcium, and magnesium

**Table 5:** Leachability results for the analyzed materials.

Element	Index						
	Białystok	Skawina	Sobieski	Staszic	Wujek	Wieczorek	Standard
pH	10.3	11.4	10.9	11.9	11.0	11.0	--- ^1^
Antimony [mg/L]	0.13	0.35	0.005	<0.005	<0.005	<0.005	PN-EN ISO 11885:2009
Arsenic [mg/L]	0.019	1.31	0.32	1.07	0.081	0.24	PN-EN ISO 11885:2009
Bar [mg/L]	0.24	0.033	0.23	0.022	0.079	0.14	PN-EN ISO 11885:2009
Chromium [mg/L]	0.19	0.032	0.19	3.96	0.19	0.023	PN-EN ISO 11885:2009
Chromium (IV) [mg/L]	0.14	0.030	0.18	3.9	0.18	0.020	PN-EN ISO 18412:2007
Zinc [mg/L]	<0.01	0.022	0.069	0.012	0.041	0.070	PN-EN ISO 11885:2009
Cadmium [mg/L]	<0.001	0.0023	<0.0005	0.0014	<0.0005	<0.0005	PN-EN ISO 11885:2009
Copper [mg/L]	<0.01	0.0051	0.0063	<0.005	<0.005	0.007	PN-EN ISO 11885:2009
Molybdenum [mg/L]	0.14	0.48	0.043	0.41	0.85	0.12	PN-EN ISO 11885:2009
Nickel [mg/L]	0.024	<0.005	0.0051	<0.005	<0.005	<0.005	PN-EN ISO 11885:2009
Lead [mg/L]	<0.005	0.0054	<0.005	<0.005	<0.005	<0.005	PN-EN ISO 11885:2009
Mercury [mg/L]	<0.001	<0.001	<0.001	<0.001	<0.001	<0.001	PN-EN ISO 12846:2012
Selenium [mg/L]	0.078	0.16	0.011	0.053	0.011	0.017	PN-EN ISO 11885:2009
Chlorides [mg/L]	12,500	56	5.3	45	5.5	5.5	PN-EN ISO 10304:2009
Fluorides [mg/L]	0.165	2.7	0.55	9.2	0.44	0.68	PN-EN ISO 10304:2009
Sulfates [mg/L]	5160	738	301	398	518	194	PN-EN ISO 10304:2009
RWO ^2^ [mg/L]	16	32	20	31	15	18	PN-EN 1484:1999
Solvent Subst.—TDS [mg/L]	31,600	4820	1480	3760	1900	1590	PN-EN 152162010

^1^ Not applicable. ^2^ Organic carbon (solved).

**Table 6:** Summary of radioactivity results for the analyzed materials.

Index	Activity Index Values		Naturally Radioactive Elements’ Concentrations		
	f_1_ [-]	f_2_ [Bq/kg]	Potassium: K^40^ [Bq/kg]	Radium: Ra^226^ [Bq/kg]	Thorium: Th^228^ [Bq/kg]
Białystok	0.25 (±0.02)	7.05 (±2.36)	625.95 (±38.30)	7.05 (±2.36)	4.08 (±1.45)
Skawina	0.9 (±0.06)	100.64 (±8.38)	573.57 (±42.97)	100.64 (±8.38)	75.25 (±5.38)
Sobieski	0.69 (±0.05)	43.00 (±5.19)	690.37 (±45.52)	43.00 (±5.19)	63.10 (±4.60)
Staszic	0.56 (±0.04)	43.41 (±5.00)	488.87 (±53.33)	43.41 (±5.00)	52.61 (±3.97)
Wujek	0.7 (±0.05)	44.85 (±5.3)	690.61 (±45.62)	44.85 (±5.3)	65.58 (±4.72)
Wieczorek	0.5 (±0.04)	28.40 (±3.97)	629.15 (±40.51)	28.40 (±3.97)	40.70 (±3.32)

**Table 7:** Results of the density and compressive strength tests.

Index	Solution	Foaming Agent	Stabilizer	Density, [kg/m^3^]	Compressive Strength [MPa]
Wieczorek	NaOH 10 M	4.5 g Al	---	1292	14.4
		1.5 g Al	---	532	0.81
		30 mL Perhydrol 36%	20 mL polyglycol	529	0.62
		30 mL Perhydrol 36%	5 g cellulose	535	0.69
Staszic		4.5 g Al	---	861	4.3
		30 mL Perhydrol 36%	20 mL polyglycol	533	0.76
		30 mL Perhydrol 36%	5 g cellulose	501	0.81

**Table 8:** Summary of selected results for thermal conductivity testing.

No.	Index	Solution	Foaming Agent	Stabilizer	Thermal Conductivity [W/(m∙K)]	Thermal Resistance [(m²∙K)/W]
1	Wieczorek	NaOH 10M	1.5 g Al		0.18522	0.1681
2	Wieczorek		30 mL Perhydrol 36%	5 g cellulose	0.11609	0.2593
3	Staszic		4.5 g Al		0.19403	0.1613
4	Staszic		30 mL Perhydrol 36%	5 g cellulose	0.15094	0.2084

## Data Availability

The original contributions presented in this study are included in the article. Further inquiries can be directed to the corresponding author.
